# EXPOsure: Milan, May 1st- October 31st 2015

**DOI:** 10.1186/s13052-015-0167-x

**Published:** 2015-08-14

**Authors:** Gian Vincenzo Zuccotti, Marco Morelli, Marco Sala, Giorgio Bedogni, Valentina Fabiano

**Affiliations:** Department of Pediatrics, Children’s Hospital V. Buzzi, Università degli Studi di Milano, via Castelvetro 32, Milan, Italy; Department of Pediatrics, Luigi Sacco Hospital, Università degli Studi di, via GB Grassi 74, Milan, Italy; Department of Pediatrics, Tradate Hospital, Piazza A. Zanaboni 1, Tradate (Varese), Italy; Liver Research Center, AREA Science Park, Strada Statale 14 - km 163.5, 34012 Basovizza, Italy

## Abstract

The city of Milan is experiencing the great event of EXPO 2015 and heavy construction has been ongoing since 2012 over an area of more than 1 million meters squared in the north-west suburban area of the city. We compared the number of hospital admissions for upper and lower respiratory tract infections (URTI and LRTI) and acute asthma, in infants and children aged between 0 and 13 years from 2011 to 2014 in two Pediatric Departments, one near and one far from the EXPO construction area. Hospital admission frequencies resulted to be similar in the two Pediatric Departments.

## Dear Editor,

It has been reported that particulate air pollution in urban areas may be responsible for human respiratory diseases [1, 2]. Children are among the most susceptible subjects.

The city of Milan is experiencing the great event of EXPO 2015 and is has undergone profound urban changes within the exhibition area. Heavy construction has been ongoing since 2012 over an area of more than 1 million meters squared in the north-west suburban area of the city. Such a large construction area is expected to produce anthropogenic emissions (particulate matters with aerodynamic diameters <2.5 mm (PM2.5) and 2.5-10 mm (PM2.5–10) possibly increasing the health risk for people living in the nearby.

We compared the number of hospital admissions for upper and lower respiratory tract infections (URTI and LRTI) and acute asthma, in infants and children aged between 0 and 13 years from 2011 to 2014, in the Pediatric Department of Luigi Sacco Hospital of Milan, the nearest hospital to the EXPO construction site, with URTI, LRTI, and asthma admissions from 2011 to 2014 in infants and children 0–13 years, in the Pediatric Department of Tradate Hospital, located in the Varese area, nearly 40 km distant to the EXPO construction site.

**Fig. 1:**
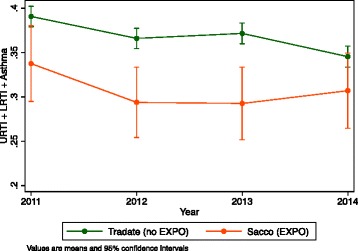
Hospital admission frequencies 2011–2014 for respiratory infections and asthma in Luigi Sacco Hospital and Tradate Hospital. URTI (Upper Respiratory Tract Infection), LRTI (Lower Respiratory Tract Infection)

Hospital admission frequencies were 34 % in 2011, 29 % in 2012, 29 % in 2013,z and 31 % in 2014 in Luigi Sacco Hospital, and 39 % in 2011, 37 % in 2012, 37 % in 2013, and 35 % in 2014 in Tradate Hospital. The trend of the number of admissions in the years 2012–2014, after the establishment of the EXPO construction site, respect to 2011, before the opening of the construction site, showed a decrease, which is comparable in both hospitals (Fig. 1).

This evidence is contrasting with an increased rate of hospital admissions for respiratory diseases in children as the result of exposure to air pollutants associated with the establishment of the EXPO construction site.

We did not conduct an air analysis for presence of particulate matters and cannot speculate on; nevertheless, these data are reassuring as regard the human health consequences possibly associated with such a great construction site.

With these data in mind, we are even more proud that Milan is the city of EXPO 2015.
